# Longitudinal clinical response to Janus kinase inhibitors in systemic sclerosis: a real-life multicentric study across multiple clinical domains

**DOI:** 10.1007/s10067-026-08168-x

**Published:** 2026-05-30

**Authors:** Stefano Di Donato, Juan José Alegre-Sancho, Anastas Batalov, Zguro Batalov, Silvia Bellando-Randone, Carmela Coccia, Marco De Pinto, Dilia Giuggioli, Michael Hughes

**Affiliations:** 1 Leeds Institute of Rheumatic and Musculoskeletal Medicine, University of Leeds, Leeds, UK; 2 ImmunoConcEpT, Université de Bordeaux, Bordeaux, France; 3 Rheumatology Unit, Hospital Universitario Dr Peset, University of Valencia, Valencia, Spain; 4 Department of Propaedeutics of Internal Diseases, Rheumatology Clinic, University Hospital “Kaspela”, Medical University of Plovdiv, Plovdiv, Bulgaria; 5 Department of Experimental and Clinical Medicine, Div. of Rheumatology, University of Florence, Scleroderma Unit, AOU Careggi, Florence, Italy; 6 Department of Medical and Surgical Sciences, Rheumatology Unit, University of Modena and Reggio Emilia, Modena, Italy; 7 Division of Musculoskeletal and Dermatological Science, School of Biological Sciences, Faculty of Biology, Medicine and Health, Centre for Musculoskeletal Research, The University of Manchester, Manchester Academic Health Science Centre, Stopford Building, Oxford Road, Manchester, UK; 8 Department of Rheumatology, Northern Care Alliance NHS Foundation Trust, Salford Care Organisation, Salford, UK; 9 NIHR Manchester Biomedical Research Centre, Manchester University NHS Foundation Trust, Manchester, UK

**Keywords:** Efficacy, JAK inhibitor, Safety, Scleroderma, Systemic sclerosis

## Abstract

**Introduction/objective:**

Janus kinase inhibitors (JAKi’s) have shown promising results in systemic sclerosis (SSc), yet little studied. We evaluated safety and intra-patient changes in pulmonary, articular, and cutaneous parameters in SSc JAKi-treated patients.

**Methods:**

An international, multi-centre, longitudinal retrospective cohort study. We assessed changes in FVC%, DLCO%, modified Rodnan skin score (mRSS), tender/swollen joint counts (TJC/SJC), digital ulcers (DU), and calcinosis. Baseline was defined as JAKi initiation. Outcomes were analysed as delta from baseline using Wilcoxon signed-rank tests, with effect sizes expressed as standardized mean change (SMC).

**Results:**

Among 32 patients treated with the four available JAKi’s, median (IQR) follow-up was 16.9 (10.3, 31.8) months, totalling 52.8 patient-years. At 12 months, pulmonary function remained stable (SMC =  + 0.22 for FVC%, + 0.23 for DLCO%; *p* = 0.10 and *p* = 0.33, respectively). mRSS (SMC =  − 0.29, *p* = 0.03), TJC and SJC (SMC =  − 1.19 and − 0.69, *p* < 0.001 and *p* = 0.001, respectively) significantly improved. At 24 months, numerical improvements in mRSS, TJC, and SJC persisted with SMC of − 0.21, − 1.13, and − 1.14 (*p* = 0.17, *p* = 0.054, *p* = 0.058, respectively). Among 11 patients with baseline calcinosis, 45.5% improved. Ten patients developed DUs. In 15 patients receiving glucocorticoids, a non-significant trend toward tapering was observed.

**Conclusion:**

JAKi treatment in SSc patients was associated with ‘real-world’ improvements in multiple domains; findings are exploratory.

**Table:** 

**Key Points** • *JAKi treatment in SSc patients was associated with ‘real-world’ improvements in multiple clinically important domains.* • *Relevant safety concerns with JAKi treatment were confirmed in our treated SSc patient cohort.* • *Our findings provide preliminary real-world evidence supporting a potential role of JAK inhibitors across multiple clinical domains in SSc.* • *Long-term controlled trials to confirm the safety and efficacy of JAKi’s in patients with SSc are needed.*

## Introduction

Systemic sclerosis (SSc) is a complex rheumatological connective tissue disease characterised by immune dysregulation, vascular dysfunction, and progressive fibrosis. Although traditionally considered a prototypical fibrotic condition, an inflammatory component is increasingly recognized in a significant subset of patients, including those with musculocutaneous and pulmonary involvement. This inflammatory phenotype (as exemplified by inflammatory arthritis, tendon friction rubs, and elevated acute phase reactants) is more frequently observed in patients with *early* diffuse cutaneous SSc (dcSSc) and is associated with a more aggressive clinical course [1], including faster progression of skin and interstitial lung disease (ILD) [2–5]. As such, immunosuppressive approaches to treatment are typically only indicated in patients with early dcSSc and/or with inflammatory manifestations [6–8].

Janus kinase inhibitors (JAKi’s) have been successfully employed in a range of immune-mediated rheumatological diseases, including rheumatoid arthritis, psoriatic arthritis, and ankylosing spondylitis. Their broad-ranging efficacy resides in the targeting of common inflammatory pathways and underpinning immune activation [9, 10]. In SSc, through modification of the JAK/STAT signalling cascade, these therapies can inhibit the transduction of multiple cytokine signals simultaneously, including interleukins (ILs) and interferons (IFNs), which are thought to drive both inflammation and fibrosis in SSc [11, 12]. Among these, type I IFN signatures have been strongly implicated in the pathogenesis of both vascular and cutaneous disease, and their downstream activation converges on the JAK/STAT axis [13–15]. Evidence of aberrant IFN pathway activation has been observed in patients with early SSc; showing predictive ability of worse disease outcomes [16, 17]. This is particularly relevant considering previous therapeutic efforts, such as the anti-IL6 receptor antibody, Tocilizumab, which showed efficacy in slowing ILD progression in SSc (but failed to meet the primary endpoint of skin score improvement) [18, 19]. This suggests that IL-6 blockade may be insufficient to modify the cutaneous trajectory of the disease, possibly due to its limited action on the IFN-driven immune component of this disease manifestation. In contrast, JAKi’s, by acting upstream and blocking IFN signalling directly, may have broader effects, potentially targeting both inflammatory and fibrotic pathways, thus avoiding therapeutic escape [20–23].

The dual anti-inflammatory and antifibrotic mechanism of action of JAKi’s makes them particularly attractive in SSc [11, 24, 25]. Nonetheless, data regarding their use in SSc remain limited to small open-label studies and case reports [26–29], and real-world evidence on both their effectiveness and safety is currently lacking. Furthermore, reports of clinical benefit observed from the treatment of idiopathic inflammatory myopathies [30, 31], including calcinosis [32], provides further therapeutic rationale for examination of JAKi in patients with SSc.

Against this background, our aim was to report the ‘real-world’ collective international experience of JAKi treatment concerning the safety and effectiveness (across multiple clinically relevant domains) in patients with SSc.

## Materials and methods

### Study design and patients

Ours was a retrospective, observational, longitudinal, multicentre cohort analysis of patients fulfilling the 2013 ACR/EULAR classification criteria for SSc [33] aged 18 or above and treated with JAKi.

Inclusion criteria were: (i) a diagnosis of SSc according to the 2013 ACR/EULAR classification criteria [33]; (ii) age ≥ 18 years; (iii) exposure to a JAK inhibitor; and (iv) availability of clinical data for at least one domain of interest (skin, joint, or lung involvement), with at least one follow-up visit after treatment initiation.

No additional formal exclusion criteria were applied. Participating centres were provided with these predefined eligibility criteria and were asked to identify consecutive eligible patients based on available data.

Clinical visits were collected using standardised case report forms and harmonised into a digital dataset for analysis. Variables reported at each timepoint were the following: cutaneous subset according to LeRoy [34], anti-nuclear antibody (ANA) positivity, presence of rheumatoid factor, anti-cyclic citrullinated peptide, anti-Ro52, anti-centromere (ACA), anti-topoisomerase I (ATA), anti-RNA polymerase III (ARA), digital pitting scars, telangiectasias, history and presence of digital ulcers (DUs), calcinosis, pulmonary arterial hypertension (PAH), ILD, glucocorticoid use, forced vital capacity (FVC) and diffusing lung capacity (DLCO) as % predicted, swollen (SJC) and tender (TJC) joint counts, presence of elevated C-reactive protein, and ongoing treatments, including conventional immunosuppressants and vasodilators.

### Ethical approval

Our study was based solely on routinely collected clinical data from patients treated as part of standard of care. All data were anonymised prior to analysis and shared between centres in accordance with local data governance regulations. Ethical approval was obtained from the principal investigator at each participating site. Anonymised data were used for analysis. Due to the retrospective and non-interventional nature of the study, formal patient consent was not required. However, patients at each centre had agreed for their clinical data to be used for future research purposes by their study team within their local institutional arrangements. The research was conducted in accordance with the Declaration of Helsinki (1964) and its later amendments.

### Study outcome definition

To assess JAKi treatment in our cohort, the following outcomes were used:

- Drug survival: defined as the time between treatment initiation and discontinuation (for any clinical reason), or the date of the last available visit.
- Clinical effectiveness: assessed through the change of the following outcomes over time: percent predicted FVC, percent predicted DLCO, modified Rodnan skin score (mRSS), tender joint count, swollen joint count, recurrence of active DUs, new onset/worsening or improvement of calcinosis.
- Glucocorticoid sparing effect: assessed by comparing the change in daily glucocorticoid dose (mg/day of prednisone or equivalent) from baseline to follow-up visits at 12 and 24 months.

### Statistical analysis

Baseline characteristics were summarized using means and standard deviation (SD), medians and interquartile range (IQR), or absolute frequencies (*n*, %) as appropriate. Patients with zero months of follow-up were excluded. For drug survival, the probability of treatment persistence was estimated using Kaplan–Meier (KM) survival curves with 95% confidence intervals. Reasons for discontinuation were tabulated separately.

For effectiveness analyses, discrete timepoints at 12 and 24 months were selected, allowing values closest to ± 3 months from these targets. The change from baseline (delta) was calculated, and the standardized mean change (SMC) was reported. Paired Wilcoxon signed-rank tests were performed to assess statistical significance. A simple linear regression model was applied to estimate follow-up changes, with baseline values included as a covariate to account for their potential confounding effect. For each of the effectiveness outcomes, a graphical smoothed linear model stratified by Leroy subset was generated. Due to the cohort follow-up, smoothed linear model representations were limited to a maximum of 36 months. A sensitivity analysis was performed excluding patients with total follow-up < 6 months to assess the robustness of the findings. Descriptive sub-analyses by individual JAK inhibitors at 12 months were performed.

The number of patients with at least one new ulcer following treatment initiation was identified and the incidence rate of active digital ulcers among the overall cohort was calculated and expressed as events per 100 patient-years. For calcinosis, we estimated the number of patients with documented presence during follow-up, and among these, the proportion who showed clinical improvement or new onset/worsening.

In patients treated with glucocorticoids, changes in dose at 12 and 24 months were assessed using paired Wilcoxon tests and SMC calculation. A linear mixed-effects model was used to estimate time-dependent trends, including random intercepts for patients. For robustness, a clustered linear regression model with sandwich standard errors was also applied. All analyses were conducted using R (version 4.3.3). The main packages used included *survival*, *survminer*, *lme4*, *ggplot2*, *lmtest*, and *sandwich*.

## Results

### Cohort characteristics and drug survival

We included 32 patients treated with the four available JAKi’s in the analysis: baricitinib (*n* = 10), filgotinib (*n* = 7), tofacitinib (*n* = 10), and upadacitinib (*n* = 5). Patients were followed up in 4 Rheumatology University Hospitals from Italy, Spain, and Bulgaria. Demographic and clinical features of the included patient cohort are presented in Table 1.

**Table 1:** Demographic and clinical features of the cohort. Continuous data are reported as medians and interquartile range. *ANA*, antinuclear antibodies; *anti*-*CCP*, anti–cyclic citrullinated peptide antibodies; *mRSS*, modified Rodnan skin score; *FVC*, forced vital capacity; *DLCO*, diffusing capacity of the lung for carbon monoxide; *CRP*, C-reactive protein

Characteristic	Overall *N* = 32^*1*^	Baricitinib *N* = 10^*1*^	Filgotinib *N* = 7^*1*^	Tofacitinib *N* = 10^*1*^	Upadacitinib *N* = 5^*1*^
Leroy subset					
Diffuse cutaneous	14 (42%)	5 (50%)	1 (14%)	5 (50%)	3 (60%)
Limited cutaneous	19 (58%)	5 (50%)	6 (86%)	5 (50%)	2 (40%)
Sex					
Female	30 (94%)	9 (90%)	7 (100%)	10 (100%)	4 (80%)
Male	2 (5.9%)	1 (10%)	0 (0%)	0 (0%)	1 (20%)
Age	61 (52, 66)	58 (53, 65)	61 (55, 71)	51 (49, 62)	63 (61, 64)
Disease duration	7 (3, 17)	7 (1, 17)	12 (6, 17)	6 (2, 13)	5 (2, 114)
Follow up (months)	17 (9, 30)	20 (15, 30)	19 (12, 36)	11 (9, 39)	5 (2, 11)
ANA positivity	32 (97%)	10 (100%)	7 (100%)	9 (90%)	5 (100%)
Rheumatoid factor	8 (24%)	3 (30%)	3 (43%)	1 (10%)	1 (20%)
Anti CCP	3 (9.1%)	1 (10%)	1 (14%)	0 (0%)	1 (20%)
Anti Ro52	2 (6.1%)	1 (10%)	1 (14%)	0 (0%)	0 (0%)
Anti centromere	8 (24%)	4 (40%)	2 (29%)	1 (10%)	1 (20%)
Anti topoisomerase I	13 (39%)	3 (30%)	1 (14%)	5 (50%)	4 (80%)
Anti RNA polymerase 3	2 (6.1%)	1 (10%)	0 (0%)	1 (10%)	0 (0%)
Pitting scars	15 (45%)	2 (20%)	3 (43%)	5 (50%)	5 (100%)
Telangiectasia	22 (67%)	8 (80%)	4 (57%)	7 (70%)	3 (60%)
History of digital ulcers	23 (70%)	5 (50%)	3 (43%)	9 (90%)	5 (100%)
Active digital ulcers	8 (24%)	0 (0%)	1 (14%)	2 (20%)	5 (100%)
Puffy fingers	17 (52%)	5 (50%)	2 (29%)	6 (60%)	4 (80%)
Calcinosis	10 (30%)	2 (20%)	3 (43%)	4 (40%)	1 (20%)
Pulmonary arterial hypertension	8 (24%)	1 (10%)	2 (29%)	3 (30%)	2 (40%)
Interstitial lung disease	17 (52%)	5 (50%)	4 (57%)	4 (40%)	4 (80%)
Glucocorticoids	15 (45%)	5 (50%)	5 (71%)	4 (40%)	1 (20%)
Anti platelet agents	8 (24%)	5 (50%)	2 (29%)	1 (10%)	0 (0%)
Mycophenolate mofetil	4 (12%)	2 (20%)	1 (14%)	0 (0%)	1 (20%)
Calcium channel blockers	12 (36%)	2 (20%)	4 (57%)	4 (40%)	2 (40%)
Endothelin receptor antagonists	8 (24%)	3 (30%)	1 (14%)	3 (30%)	1 (20%)
Phosphodiesterase 5 inhibitors	11 (33%)	0 (0%)	1 (14%)	5 (50%)	5 (100%)
Iloprost	7 (21%)	1 (10%)	5 (71%)	0 (0%)	1 (20%)
Methotrexate	12 (36%)	3 (30%)	2 (29%)	4 (40%)	3 (60%)
Leflunomide	2 (6.1%)	0 (0%)	0 (0%)	1 (10%)	1 (20%)
Tocilizumab	1 (3.0%)	0 (0%)	0 (0%)	1 (10%)	0 (0%)
Nintedanib	1 (3.0%)	1 (10%)	0 (0%)	0 (0%)	0 (0%)
Rituximab	2 (6.1%)	1 (10%)	0 (0%)	0 (0%)	1 (20%)
mRSS	7 (2, 13)	2 (0, 10)	6 (1, 14)	10 (7, 12)	8 (7, 23)
FVC%	92 (79, 108)	97 (91, 108)	95 (79, 135)	91 (78, 97)	84 (76, 93)
DLCO%	71 (58, 84)	69 (56, 80)	66 (56, 70)	85 (63, 95)	81 (65, 90)
Swollen joint count	4.0 (2.0, 10.0)	4.0 (2.0, 8.0)	3.0 (0.0, 10.0)	6.0 (2.0, 10.0)	4.0 (0.0, 8.0)
Tender joint count	6.0 (2.0, 10.0)	5.0 (2.0, 8.0)	8.0 (5.0, 11.0)	6.0 (2.0, 13.0)	3.5 (0.0, 10.0)
Increased CRP	10 (30%)	4 (40%)	2 (29%)	3 (30%)	1 (20%)

^*1*^ *n* (%); median (Q1, Q3)

The median (IQR) age was 60 (54, 64) years, with 29 (91%) female and 31 (97%) ANA positive patients. Nineteen patients (59%) presented with the limited cutaneous and 13 (41%) with the diffuse cutaneous subsets according to LeRoy classification. Sixteen (50%) patients presented ILD at baseline, and 8 (25%) had a right-heart catheter proven diagnosis of PAH, 22 (69%) patients had a history of DUs, and 7 (22%) presented an active DU at baseline visit.

The baseline median (IQR) FVC% and DLCO% predicted were 92% (78, 108) and 71% (57, 84), respectively. Twelve (38%) patients were on methotrexate, 3 (9.4%) were on mycophenolate mofetil, 2 (6.3%) were on leflunomide, and 1 (3.1%) on rituximab. Fifteen (47%) patients were on concomitant glucocorticoid treatment, as detailed in Table 1. Disease features and SSc-specific treatments are reported in Table 1.

**Fig. 1:**
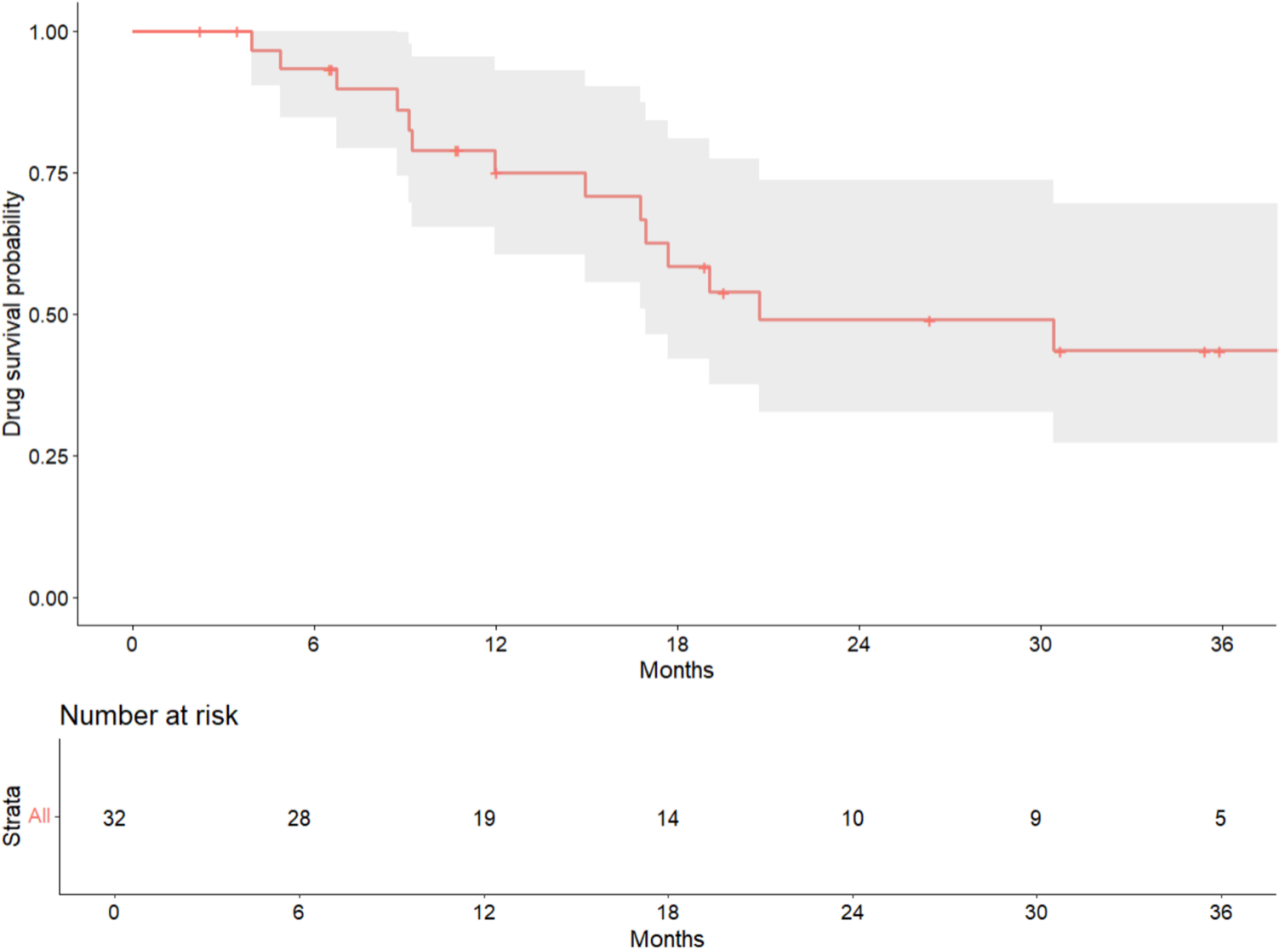
Kaplan–Meier curve for the overall cohort representing the drug survival probability over time in SSc patients treated with JAKi’s

The median (IQR) follow-up time on treatment was 16.9 (10.3–31.8) months, with a range from 1.4 to 52.0 months, amounting to 52.8 patient-years. During follow up, 17 patients suspended JAKi treatment, 7 due to primary ineffectiveness (either on cutaneous, articular, or calcinotic involvement), 3 due to secondary failure, 3 due to severe infections, 1 due to cancer onset (lung carcinoma), 1 due to acute kidney failure (not attributable to scleroderma renal crisis but rather to biopsy-proven interstitial nephritis in the context of concomitant multi-therapy and not directly linked to JAKi use), 1 due to severe leukopenia, and 1 due to recurrent aphthous ulcerations. Kaplan–Meier drug survival curve is reported in Fig. 1.

### Effectiveness endpoints

#### Forced vital capacity and diffusing lung capacity change

**Fig. 2:**
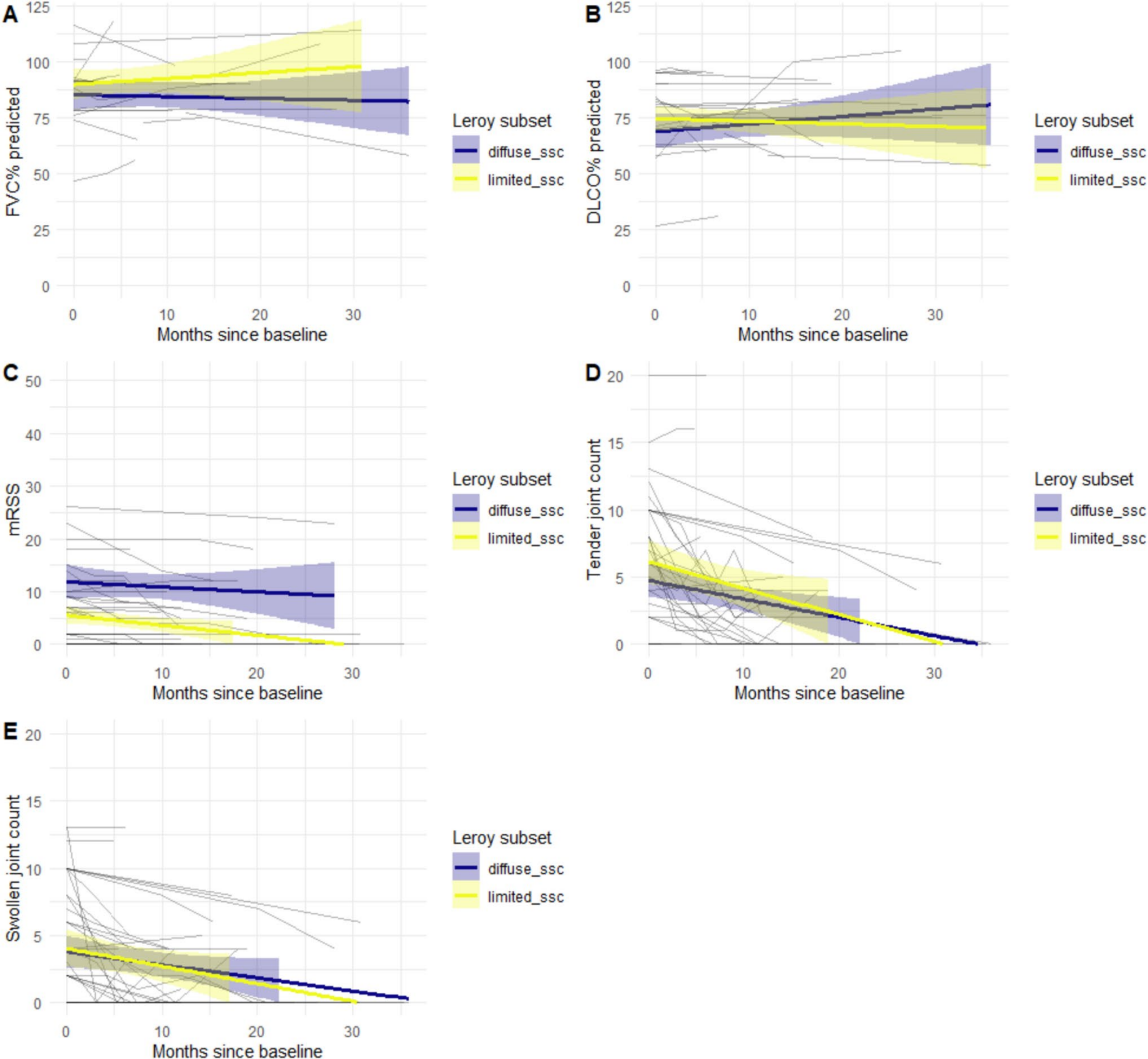
Longitudinal changes in clinical parameters during JAK inhibitor treatment in systemic sclerosis. **A** Forcev Vital Capacity (FVC) % predicted, **B** Diffusing Lung Capacity (DLCO) % predicted, **C** modified Rodnan skin score (mRSS), **D** tender joint count (TJC), and **E** swollen joint count (SJC), stratified by LeRoy subset. Each line represents an individual patient; solid lines and shaded areas represent linear trends with 95% confidence intervals

At 12 months, FVC% predicted showed no significant deterioration compared to baseline with a small, numerically positive SMC of + 0.22 (*p* = 0.10). DLCO% predicted showed a similar trend at the 12-month mark, with no significant deterioration and a SMC of + 0.23 with no statistical significance (*p* = 0.33) (Fig. 2A and B). At 24 months, data on pulmonary function were available for only 8 patients: compared to baseline, no significant change was observed for FVC with a SMC of + 0.26 (*p* = 0.25) and for DLCO with a SMC of + 1.27 (*p* = 0.59).

### Modified Rodnan skin score change

At 12 months, the median mRSS showed a significant improvement with an SMC of − 0.29 (*p* = 0.03) and higher baseline mRSS predicted a greater reduction of mRSS at follow-up (*β* =  − 0.316, *p* = 0.009) (Fig. 2C). At 24 months (*n* = 8), no significant change was observed, with only a numerical improvement of mRSS (SMC =  − 0.210, *p* = 0.174). In a linear regression model adjusted for disease duration, baseline mRSS showed a trend toward association with change over time (*β* =  − 0.253, *p* = 0.058), while disease duration was not significantly associated with mRSS change (*β* =  − 0.034, *p* = 0.40).

### Joint involvement change

Twenty-nine patients (91%) presented with joint involvement at baseline; of these, 12 (41%) had raised baseline C-reactive protein levels. TJC significantly improved at 12 months (SMC =  − 1.19; *p* < 0.001), with baseline TJC predicting the effect size (*β* =  − 0.576, *p* = 0.001). Likewise, SJC significantly improved at 12 months (SMC =  − 0.693, *p* = 0.001), with greater reduction in patients with higher baseline counts (*β* =  − 0.640, *p* < 0.001) (Fig. 2D). At 24 months (*n* = 8), a favourable trend persisted for SJC improvement (SMC =  − 1.143, *p* = 0.058), with higher baseline counts again approaching significance in predicting articular response (*β* =  − 0.781, *p* = 0.10) (Fig. 2E).

### Sensitivity analysis

In sensitivity analyses restricted to patients with ≥ 6 months follow-up (*n* = 28), the magnitude and direction of effect sizes at 12 months were consistent with the primary analysis, with persistent improvement in mRSS, TJC, and SJC, while pulmonary function parameters remained unchanged (Table 2). Analyses at 24 months are reported for descriptive purposes only due to limited sample size (Table 2).

**Table 2:** Sensitivity analysis restricted to patients with ≥ 6 months follow-up (*n* = 28). *FVC*, forced vital capacity; *DLCO*, diffusing capacity of the lung for carbon monoxide; *mRSS*, modified Rodnan skin score

Outcome	Standardized mean change (12 months)	*p*-value (12 months)	Standardized mean change (24 months)	*p*-value (24 months)
FVC (%)	0.21	0.102	0.76	0.181
DLCO (%)	0.24	0.330	0.97	0.586
mRSS	− 0.29	**0.030**	− 0.21	0.174
Tender joint count	− 1.19	**0.0002**	− 1.13	0.054
Swollen joint count	− 0.69	**0.0012**	− 1.14	0.058

Descriptive analyses stratified by individual JAK inhibitors are reported at 12 months (Table 3).

**Table 3:** Subanalysis by JAK inhibitor type. *p*-values are not adjusted for multiple comparisons. Analyses for upadacitinib were not estimable due to insufficient number of patients with available paired data at the specified timepoint. *FVC*, forced vital capacity; *DLCO*, diffusing capacity of the lung for carbon monoxide; *mRSS*, modified Rodnan skin score

JAK inhibitor	Outcome	Standardized mean change (12 months)	*p*-value
**Baricitinib**	FVC (%)	0.65	0.058
	DLCO (%)	0.36	0.418
	mRSS	− 0.04	0.773
	Tender joint count	** − 1.20**	**0.034**
	Swollen joint count	− 1.02	0.058
**Tofacitinib**	FVC (%)	− 0.23	1.000
	DLCO (%)	0.61	1.000
	mRSS	− 0.08	1.000
	Tender joint count	− 0.78	0.174
	Swollen joint count	− 0.60	0.149
**Filgotinib**	FVC (%)	0.16	0.402
	DLCO (%)	0.19	0.675
	mRSS	− 0.62	0.181
	Tender joint count	** − 2.30**	**0.034**
	Swollen joint count	− 0.56	0.181

### Calcinosis cutis and digital ulcers

Among 11 patients with calcinosis at baseline, 5 (46%) showed improvement, 3 (27%) experienced worsening, and 3 (27%) remained stable during follow-up. During follow-up, 10 patients developed at least one episode of active DUs, corresponding to an incidence rate of 21 events per 100 patient-years.

### Glucocorticoid sparing effect

Among patients treated with glucocorticoids (*n* = 15), a trend toward dose tapering over time was observed (− 0.038 mg/month, *p* = 0.1). At subject level, 13 out of 15 patients showed a dose reduction of glucocorticoid use during follow-up. Precisely, at 12-month follow-up, a baseline mean dose of 6.9 mg/day decreased to 2.3 mg/day, with a SMC of − 0.91 (*p* = 0.069). Due to insufficient paired data at 24 months, no formal analysis was performed on glucocorticoid dose evolution at this timepoint.

## Discussion

We present results from an international ‘real-world’ cohort of SSc patients treated with JAKi’s, where we observed a favourable impact on several clinically relevant (lung, skin, and joints) domains. We also report experience concerning significant improvements observed with calcinosis in many patients, and a trend toward successful facilitation of glucocorticoid tapering over time.

A significant improvement in mRSS was observed at 12 months, particularly in those patients with higher baseline scores, and this effect persisted numerically at 24 months although this latter observation is based on a very limited number of patients and should be interpreted as descriptive only. The association between baseline mRSS and its change over time was attenuated after adjustment for disease duration, suggesting that part of this relationship may be explained by baseline severity and statistical effects such as regression to the mean. Importantly, disease duration itself was not associated with mRSS change, supporting that the observed improvement was not driven by differences in disease chronicity. Notably, the cutaneous response emerged despite the predominance of limited cutaneous subset in our cohort (~ 60%), which could have attenuated the observable effect size. This contrasts with clinical trials that typically enrol patients with diffuse cutaneous involvement, where skin improvement may be more pronounced by design. Therefore, the observed effectiveness in our real-world population with a broader disease spectrum further strengthens the clinical relevance of this observation. However, an additional point to consider is the potential influence of regression to the mean. The observed association between higher baseline values and greater magnitude of improvement across several clinical domains (including mRSS) may, at least in part, reflect this statistical phenomenon, which is inherent to pre–post analyses in the absence of a control group. Therefore, these findings should not be interpreted as evidence of preferential treatment efficacy in patients with more active disease. Nevertheless, the consistency of improvement across multiple independent clinical outcomes suggests that regression to the mean alone is unlikely to fully account for the observed changes, although this possibility cannot be formally excluded in the absence of a comparator arm.

Similar to the clinical benefit observed with skin involvement, pulmonary function remained overall stable over time, with small numerical improvements in both FVC and DLCO at 12 and 24 months. Although this was not statistically significant, this trend suggests a potential stabilizing effect on lung involvement, which is a key therapeutic objective in SSc. However, the small number of patients with pulmonary follow-up data at 24 months (*n* = 8) severely limits the interpretability of these findings, which should be considered descriptive only rather than inferential.

Likewise, both tender and swollen joint counts improved significantly, with large effect sizes and a predictive value of baseline activity, indicating that JAKi’s may be particularly beneficial in patients with inflammatory joint manifestations. This is clinically relevant, as patients with articular manifestations have been reported to be associated with more severe disease phenotypes [1], often accompanied by elevated CRP levels, a feature also observed in our cohort. Notably, both skin and joint involvement are recognized as IFN-driven domains in SSc [35, 36] and other connective tissue diseases such as Systemic Lupus Erythematosus [37–39], and SSc patients with high interferon signatures have been shown to experience worse outcomes. In this regard, the ability of JAKi to modulate this pathway may explain our cutaneous and articular findings and be particularly advantageous in this subset of patients with immunologically active and clinically aggressive disease, as shown in our cohort with a high prevalence of ATA antibodies (38%), rheumatoid factor (22%) and anti-CCP (7%), alongside elevated CRP levels in nearly one third of patients. Glucocorticoid use was largely driven by inflammatory musculoskeletal involvement, particularly arthritis, consistent with the observed improvement in joint-related outcomes. However, specific indications for glucocorticoid use were not systematically recorded, and therefore the interpretation of steroid-sparing effects should be made with caution.

Remarkable improvements were observed concerning apparent benefit for calcinosis, which is a difficult-to-treat manifestation of the disease, with no known disease-modifying therapy [37]. Nearly half of the patients with calcinosis at baseline experienced improvement. Furthermore, the incidence rate of new DU events remained relatively low (21/100 patient-years) [38–40]. This was despite the vascular phenotype of our population, characterized by 22% of patients with active DUs at baseline and ~ 70% with a history of DUs. Published data indicate that 50–70% of SSc patients experience DUs and around 10% develop new DUs annually, supporting the plausibility of our findings [41,42,43] However, these observations remain exploratory and do not allow definitive attribution of a causal treatment effect in the absence of a control group.

Drug safety and persistence were important aspects in our real-word cohort of patients with SSc treated with JAKi. Specifically, drug discontinuation occurred in half (53%) of patients over a median of 17 months, with one third of cases due to lack of efficacy, and others (28%) due to safety concerns, most commonly infections or hematologic adverse events. These data highlight the need for longer-term safety data specific to the SSc patient population, which may differ from other diseases (e.g., due to differences in concomitant medications and/or internal organ involvement). Importantly, despite known FDA safety warnings regarding major adverse cardiovascular events and thromboembolic risks associated with JAKi’s, no such events were reported in our cohort, and no worsening of pre-existing PAH was observed. This finding is particularly relevant in the context of SSc, wherein vascular complications are a key concern.

Our study provides important insights for clinical practice but has several limitations. The retrospective, non-controlled design limits causal inference, and the small sample size at later timepoints reduces the statistical power, particularly for pulmonary and vascular endpoints where the effect size appears to be limited compared to cutaneous and articular domains. Our cohort included different JAK inhibitors with distinct selectivity profiles and pharmacological properties. Although descriptive analyses stratified by individual agents showed broadly consistent trends, these findings are limited by small sample size and should be interpreted with caution. Therefore, potential differences between individual JAK inhibitors could not be reliably assessed in this study. In addition, no formal primary endpoint was pre-specified and multiple outcomes were evaluated in parallel. As such, all analyses should be considered exploratory in nature. The absence of adjustment for multiple comparisons increases the risk of type I error, and therefore statistically significant findings (including those observed for mRSS) should be interpreted with caution. Furthermore, the number of patients contributing data decreased over time due to attrition inherent to real-world longitudinal follow-up. Although data were complete for the variables analysed at each visit, this progressive reduction in sample size may introduce bias, including informative censoring, as patients discontinuing treatment or with shorter follow-up may differ systematically from those remaining under observation. To this matter, at the 24-month timepoint, analyses were based on a very limited number of patients which substantially limits the stability and interpretability of statistical estimates for longer follow-up. Therefore, findings at this timepoint should be considered descriptive only and not suitable for inferential conclusions. Moreover, detailed longitudinal data on treatment modifications were not consistently available across centres. Therefore, potential changes in background therapy during follow-up may have acted as uncontrolled confounders and influenced the observed clinical outcomes.

Lastly, data were collected from a limited number of centres—with over 50% of patients enrolled in Italy—which may limit the generalizability of our findings to broader international SSc populations. Ours was a pragmatic study which leveraged routinely collected clinical data, and the lack of biomarkers or imaging data precludes further mechanistic interpretation. Importantly, identifying the molecular profile of patients who are more likely to respond or fail to respond to JAK/STAT inhibition remains an important question. Such stratification could guide personalized treatment strategies and optimize therapeutic outcomes in this heterogeneous disease.

JAKi treatment in SSc patients was associated with ‘real-world’ improvements in multiple clinically important domains. However, relevant safety concerns with JAKi treatment were confirmed in our collectively treated SSc patient cohort. Despite these limitations, our findings suggest a potential role for JAK/STAT pathway inhibition across multiple disease domains in selected SSc patients. Prospective controlled trials with adequate follow-up and mechanistic exploratory endpoints are warranted to confirm the efficacy, safety, and ideal patient profiles for JAKi therapy in SSc.

## Data Availability

Sharing of the data underlying this article will be considered on reasonable request to the corresponding author.
